# Laryngeal Compartmentalization Does Not Affect the Prognosis of T3-T4 Laryngeal Cancer Treated by Upfront Total Laryngectomy

**DOI:** 10.3390/cancers12082241

**Published:** 2020-08-11

**Authors:** Filippo Marchi, Francesco Missale, Claudio Sampieri, Marta Filauro, Andrea Iandelli, Giampiero Parrinello, Fabiola Incandela, Ludwig E. Smeele, Michiel W. M. van den Brekel, Francesca Del Bon, Piero Nicolai, Cesare Piazza, Giorgio Peretti

**Affiliations:** 1 IRCCS Ospedale Policlinico San Martino, 16132 Genoa, Italy; filippomarchi@hotmail.it (F.M.); missale.francesco@gmail.com (F.M.); mfilauro@yahoo.com (M.F.); iandelliandrea@gmail.com (A.I.); giampiero.parrinello@gmail.com (G.P.); giorgioperetti18@gmail.com (G.P.); 2 Department of Surgical Sciences and Integrated Diagnostics (DISC), University of Genoa, 16132 Genoa, Italy; 3 Department of Plastic and Reconstructive Surgery, Chang Gung Memorial Hospital, Taipei 33305, Taiwan; 4 Department of Molecular and Translational Medicine, University of Brescia, 25100 Brescia, Italy; 5 Department of Experimental Medicine (DIMES), University of Genoa, 16132 Genoa, Italy; 6 Department of Head and Neck Surgery, Chang Gung Memorial Hospital, Chang Gung University and Medical College, Taoyuan 33305, Taiwan; 7 Department of Otorhinolaryngology, Maxillofacial and Thyroid Surgery, Fondazione IRCCS, National Cancer Institute of Milan, 20133 Milan, Italy; Fabiola.Incandela@istitutotumori.mi.it (F.I.); ceceplaza@libero.it (C.P.); 8 Department of Head & Neck Oncology & Surgery Otorhinolaryngology, Antoni Van Leeuwenhoek, Nederlands Kanker Instituut, 1066 Amsterdam, The Netherlands; l.smeele@nki.nl (L.E.S.); m.vd.brekel@nki.nl (M.W.M.v.d.B.); 9 Department of Otorhinolaryngology—Head and Neck Surgery, University of Brescia, 25123 Brescia, Italy; delbonfrancesca@gmail.com; 10 Section of Otorhinolaryngology—Head and Neck Surgery, Department of Neurosciences, University of Padua, Via Giustiniani, 2-35128 Padua, Italy; pieronicolai@icloud.com; 11 Department of Oncology and Oncohematology, University of Milan, 20122 Milan, Italy

**Keywords:** laryngeal neoplasms, total laryngectomy, paraglottic space, tumor thickness, prognosis, multicentric study

## Abstract

A picture is emerging in which advanced laryngeal cancers (LCs) are potentially not homogeneous and may be characterized by subpopulations which, if identified, could allow selection of patients amenable to organ preservation treatments in contrast to those to be treated with total laryngectomy (TL). This work aims to analyze a multicentric cohort of T3-T4a LCs treated by upfront TL, investigating the clinical and pathological features that can best predict oncologic outcomes. A total of 149 previously untreated patients who underwent TL for T3-T4a LC at four institutions were analyzed. Survival and disease-control were considered as the main outcomes. A secondary end-point was the identification of covariates associated with nodal status, investigating also the tumor thickness. T and N categories were significantly associated with both overall and disease-specific survival. The number of positive nodes and tracheal involvement were associated with loco-regional failure; post-cricoid area invasion and extra-nodal extension with distant failure. Posterior laryngeal compartment involvement was not a significant prognostic feature, by either univariable and multivariable analyses. These results support the conclusion that laryngeal compartmentalization has no impact on survival in patients treated by upfront TL and the current TNM staging system remains a robust prognosticator in advanced LC.

## 1. Introduction

Laryngeal cancer (LC), based on the Surveillance, Epidemiology, and End Results (SEER) database for the period 2009–2015, accounts for 0.7% of all new malignant tumors diagnosed in the United States each year. The estimated 5-year overall survival (OS) rate, considering all stages, is 60.3% and has not changed appreciably over the past several decades [1,2,3]. Moreover, a majority of patients are still diagnosed with locally advanced (T3-T4) disease and evidence of regional nodal metastases, with survival rates generally inferior to 50% [4]. During recent decades, two milestone studies have demonstrated that organ preservation (OP) was achievable even for advanced LC using non-surgical strategies [5,6]. Consequently, chemoradiation (CRT) has become increasingly popular, causing a therapeutic paradigm shift from upfront total laryngectomy (TL) to concurrent/induction CRT. Nevertheless, several epidemiologic studies have reported a decline in survival for patients with LC, possibly attributable to the indiscriminate use of OP protocols [2]. In response to this, the latest American Society of Clinical Oncology (ASCO) position paper, recommendation 2.2, points out that “for selected patients with extensive T3 or large T4a lesions and/or poor pre-treatment laryngeal function, better survival rates and quality of life may be achieved with TL rather than with OP approaches and may be the preferred treatment” [4].

Indeed, the standard of care for intermediate-advanced LC is still a matter of international debate. To furtherly complicate this issue, the 8th Edition of the AJCC UICC TNM staging system includes a wide gamut of different lesions under the generic label of locally advanced LC, ranging from T3 with minimal vs. massive paraglottic space (PGS) involvement (with normal or impaired/fixed vocal cord and arytenoid mobility), T3 with pre-epiglottic space (PES) infiltration, T3 with inner cortex thyroid cartilage erosion, T4 with full-thickness infiltration of the laryngeal framework, and/or T4 with extra-laryngeal extension [7]. In fact, contemporary endoscopic [8] and radiologic [9,10] work-up have dramatically reduced the diagnostic uncertainty during pre-treatment evaluation, leading to better profiling of advanced LC subcategories, and allowing a more tailored treatment choice for each in terms of oncological and functional outcomes.

For example, regarding open partial horizontal laryngectomies (OPHLs) applied to T3-T4 LCs, some authors have recently shown that tumor extension (distinguished in anterior vs. posterior PGS involvement, based on a virtual coronal plane passing through the arytenoid vocal process and perpendicular to the ipsilateral thyroid lamina) is a major prognosticator [11,12]. They therefore concluded that anterior cT3 tumors, without arytenoid fixation, can be successfully managed by OPHLs, and this approach could also be proposed for treatment of anterior cT4aN0, whereas it should be considered less safe in posterior cT3 tumors with crico-arytenoid joint fixation, since these lesions have clinical and biological behaviors that are quite similar to cT4a tumors. As a consequence, a picture is gradually emerging in which advanced LCs appear to be not non-homogeneous, but rather consist of different subpopulations which, if identified, could allow selection of patients who respond better to OP treatments, in contrast to those who should be immediately selected for upfront TL.

With this work we tried to verify if laryngeal compartmentalization may be the result of a different biological behavior of LC or, if instead, it is only a treatment-related prognosticator. Accordingly, we investigated if this feature still impacts on prognosis when applying a more aggressive surgical treatment as TL. Moreover, we evaluated the survival impact of each clinical, radiological, and pathological feature that defines LC as “advanced”, including different tumor extensions to the visceral PGS and PES, pathways of spread, infiltration of the laryngeal framework, involvement of extra-laryngeal soft tissues, and TNM stage. In addition, we investigated the role of promising prognosticators such as tumor thickness (TT) and number of positive nodes. We believe that these findings may allow physicians to better target some of the most relevant prognostic determinants in the advanced LC scenario, thus helping in the difficult aim of maximizing both OP and oncologic cure rates.

## 2. Materials and Methods

Between January 2010 and September 2018, a total of 169 patients affected by advanced LC who underwent upfront TL were enrolled at four different centers. These included the Unit of Otorhinolaryngology—Head and Neck Surgery of the Ospedale Policlinico San Martino, Genoa, Italy (N = 40), ASST Spedali Civili, Brescia, Italy (*N* = 78), National Cancer Institute, Milan, Italy (N = 31), and the Nederlands Kanker Instituut, Amsterdam, The Netherlands (N = 20). The study was conducted in accordance with the Declaration of Helsinki, and the study was approved by the local Ethics Committee (CER Liguria: 230/2019). All patients had been submitted to surgery after multidisciplinary team (MDT) discussion and preoperative counseling between head and neck surgeons, and radiation and medical oncologists. Patients were selected for upfront TL if not amenable to OP strategies, either surgical (transoral laser microsurgery (TLM), or OPHL) and non-surgical CRT protocols [13]. Other selection criteria for inclusion this retrospective analysis were: (1) no history of previous LC; (2) no history of previous laryngeal treatments; (3) availability of imaging and endoscopies performed no more than 4 weeks before surgery; (4) final histopathologic report confirming squamous cell carcinoma (SCC); (5) and final pathologic staging of pT3-pT4a LC.

All data concerning comorbidities, preoperative staging, surgical outcomes, histopathology, adjuvant therapies, and follow-up were collected in a single dedicated database. Preoperative work-up was standardized for all patients and consisted of endoscopic and imaging evaluation. The endoscopic work-up included preoperative transnasal videolaryngoscopy to assess vocal fold/arytenoid mobility, and intraoperative rigid endoscopy by 0°, 30°, and 70° telescopes with white light (WL) and narrow band imaging (NBI, Olympus Medical System Corporation, Tokyo, Japan) to better define the superficial extension of the lesion [8]. Either computed tomography (CT) or magnetic resonance (MR) were used for preoperative imaging. Neck ultrasound (US) with or without fine-needle aspiration cytology was routinely performed. The radiologic work-up allowed to meticulously assess 3D tumor extension, the entity of PGS and/or PES involvement, the presence of cartilaginous framework transgression, and invasion of extra-laryngeal soft tissues [14]. Tumors were classified according to the 8th Edition of the AJCC UICC TNM staging system [7].

PGS involvement was retrospectively re-assessed according to previously published criteria, considering a frontal plane passing through the arytenoid vocal process and perpendicular to the ipsilateral thyroid lamina as the boundary between anterior vs. posterior PGS [10,11,12]. Laryngeal motility and involvement of the medial wall of the piriform sinus were also considered as ancillary signs for the definition of anterior vs. posterior topography of each tumor (T-topography). Therefore, tumors were considered to involve the posterior laryngeal compartment when posterior PGS was radiologically invaded and/or the arytenoid was fixed and/or medial wall of the piriform sinus was entirely involved.

The main histopathological features considered in the present analysis were: status of surgical margins (close margins defined as <1 mm), perineural invasion (PNI), lympho-vascular invasion (LVI), number of lymph nodes involved, and extra-nodal extension (ENE). Further features regarding the pathways of diffusion of each tumor were also considered; these encompassed: laryngeal framework infiltration or extension to the tracheal rings, soft tissues of the neck, thyroid gland, and base of tongue or retro-cricoid area. Moreover, in 68% of patients in our cohort, TT, derived from microscopic measurements within the histopathologic specimen, was also available and studied in relation to nodal status.

### 2.1. Treatments and Follow-Up

All patients underwent TL or TL combined with partial pharyngectomy (PPH). Selective (SND) or modified radical neck dissections (MRNDs) were performed in adherence with National Comprehensive Cancer Network (NCCN) guidelines for cT3 and cT4 LC [15].

Postoperative radiotherapy (PORT) was discussed by the MDT board and proposed based on pathological findings of pT4a category, close or positive resection margins, nodal category ≥pN2a, and presence of PNI and/or LVI in the final histopathologic report, according to NCCN guidelines [15]. PORT dose ranged from 54 to 66 Gy. Platinum-based chemotherapy (CHT) was offered in combination with RT to all patients with positive resection margins or ENE, taking into account the patients’ age and comorbidities.

Follow-up was performed by clinical examination of the remaining upper aero-digestive tract and neck and, according to European Laryngological Society (ELS) guidelines, included CT/MR and/or PET-CT if indicated by clinical doubts after endoscopic evaluation [16]. The mean follow-up time in the present cohort was 36 months (range, 6–180).

### 2.2. Statistical Analysis

Standard descriptive statistics was used for data summarizing. For group comparisons in qualitative variables, the Chi-square test was applied. Survival analysis, considering as outcomes overall (OS), disease-specific (DSS), loco-regional recurrence-free (LRRFS), and distant recurrence-free survivals (DRFS), was performed with uni- and multivariable Cox proportional-hazards models. OS was defined as the time between the date of surgery and date of death/last visit, DSS as the time between the date of surgery and date of cancer-related death/last visit, LRRFS as the time between the date of surgery and date of local or nodal recurrence/last visit, and DRFS as the time between the date of surgery and date of distant recurrence/last visit.

Cut-offs in continuous predictors were estimated using maximally selected log-rank statistics [17]. Uni- and multivariable logistic regressions were built considering the presence of positive lymph nodes as the dependent variable. Multivariable Cox models were built by stepwise covariates selection keeping T-topography as an investigative variable inside all multivariable models. Survival estimates were reported as hazard ratio (HR) with 95% confidence interval (95% CI) and estimating the 2- and 5-year survival probability with 95% CI for variables of main clinical interest. Univariable survival curves were plotted by the Kaplan–Meier method and compared with the Log-rank test. In all analyses, two-tail tests with a significance level of 5% were applied. Stata (version 13.0, College Station, TX, USA) and R (version 3.5.1, R Foundation for Statistical Computing, Vienna, Austria) were used for statistical analysis.

## 3. Results

### 3.1. Demographics

A total of 169 patients met the inclusion criteria for retrospective analysis. Due to missing data, only 149 were available for statistical analysis. Mean age was 67 years (range, 41–92), while there were 126 males (84.6%) and 23 females (15.4%). A total of 142 (95.3%) patients were or had been smokers, and 75 (50.3%) were routine alcohol consumers.

### 3.2. Treatments and Tumor Features

In total, 60 (40.3%) patients underwent TL or TL with PPH alone, while 72 (48.3%) received PORT and 17 (11.4%) adjuvant CRT. A total of 112 (75.2%) received a bilateral neck dissection, 34 (22.8%) a unilateral one, and three (2%) received none due to major medical comorbidities.

A total of 138 tumors were located in multiple laryngeal sites (92.6%), whereas six (4%) were confined to the supraglottis, four (2.7%) involved the glottis alone, and one (0.7%) was a purely subglottic tumor.

At the final histopathologic report, 69 (46.3%) tumors were classified as pT3, and 80 (53.7%) as pT4a. In total, eight (5.4%), 89 (59.7%), and 52 (34.9%) tumors were described as well-, moderately-, and poorly-differentiated SCCs, respectively. Furthermore, 75 (43.6%) patients had nodal metastases (11 pN1, 10 pN2a, 14 pN2b, 3 pN2c, and 28 pN3b). In particular, pathologic ENE was found in 38 (25.5%) patients, PNI in 80 (53.7%), and LVI in 74 (49.7%). Positive margins were reported in 11 (7.4%) patients, while close margins were present in 19 (12.8%). Thyroid cartilage was partially invaded in 30 (20.1%) cases, and involved with full-thickness in 35 (23.5%). Cricoid cartilage was infiltrated in 36 (24.2%) and tumors reached the tracheal rings in eight (5.4%). Soft-tissue extra-laryngeal extension was confirmed in 50 (33.6%) specimens. Pathologic TT was reported in 102 (68.4%) patients, with a mean value of 14.6 mm (range, 1.1–31).

Regarding T-topography, tumors were considered posteriorly compartmentalized when the posterior PSG was radiologically invaded, and/or the arytenoid was fixed, and/or the medial wall of the piriform sinus was entirely involved. As a result of this categorization, they were divided in 19 (12.7%) anterior pT3 tumors, 50 (33.6%) posterior pT3, 21 (14.1%) anterior pT4a, and 59 (39.6%) posterior pT4a. Full clinical and pathological data are reported in Table 1.

### 3.3. Survival Estimates

Median OS was 82 months, with a mean follow-up time of 36 months (range, 6–180). At the last follow-up (November 2019), 45 (30.2%) patients had died: 28 (18.8%) for disease progression, and 17 (11.4%) for other causes. The remaining 104 (69.8%) were alive with no evidence of disease. A total of 47 recurrences were recorded, 26 of which were diagnosed at distant sites and 21 loco-regional. Seven patients experienced both loco-regional and distant recurrences. In total, 10 of the recurrences were treated surgically with curative intent, while 7 were managed by palliative RT, 15 with palliative CHT, and 15 by best supportive care.

The 2- and 5-year OS rates for the entire cohort were 77% and 63%, respectively, while 2- and 5-year DSS rates were 84% and 75%. Further details of 2- and 5-year survival estimates are summarized in Table S1.

### 3.4. Univariable Survival Analysis

End-points considered for uni- and multivariable survival analyses with Cox-proportional hazards models were OS, DSS, LRRFS, and DRFS. For all outcomes analyzed, pT category (pT4a vs. pT3), pN category, histopathologic evidence of ENE, number of positive nodes, and involvement of the retro-cricoid area were significant predictors at univariable analysis, as reported in Table 2; Table 3. Regarding OS and DSS, the full-thickness involvement of thyroid cartilage (*p* = 0.008 and *p* = 0.006, respectively), presence of extra-laryngeal extension (*p* = 0.018 and *p* = 0.16, respectively), and need for adjuvant therapy (*p* < 0.05 for both) were also adverse prognostic features at univariable analysis (Table 2).

Conversely, tracheal (*p* = 0.001) and retro-cricoid area involvement (*p* = 0.040) were associated with worse LRRFS, while close margins (*p* = 0.006), need for adjuvant CRT (*p* = 0.011), tracheal involvement (*p* = 0.036), and LVI (*p* = 0.032) were associated with worse DRFS at univariable analysis (Table 3). Of note, the anterior vs. posterior laryngeal compartmentalization was not associated with different oncologic outcomes (Tables S2 and S3). Further details of univariable survival analysis are reported in Tables S1 and S3 and Figure S1.

### 3.5. Multivariable Survival Analysis

By a stepwise covariate selection, keeping T-topography as an investigative variable inside all multivariable models, a Cox proportional hazards model for each oncologic outcome was built.

**Figure 1:**
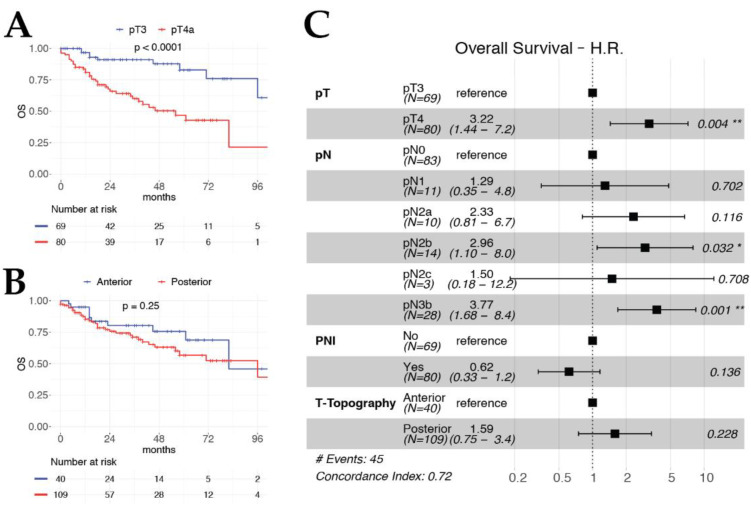
Kaplan–Meier curves for OS according to pT category (**A**) and T-topography (**B**). (**C**) Forest plot of OS multivariable model.

**Figure 2:**
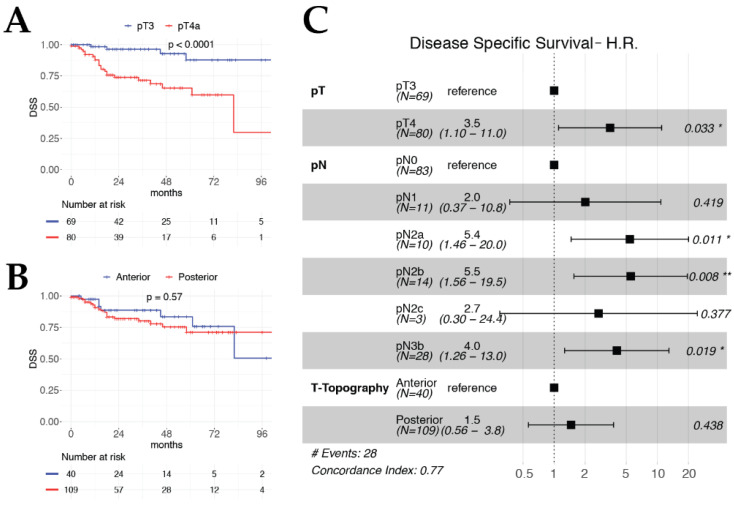
Kaplan–Meier curves for DSS according to pT category (**A**) and T-topography (**B**). Forest plot of DSS multivariable model (**C**).

**Figure 3:**
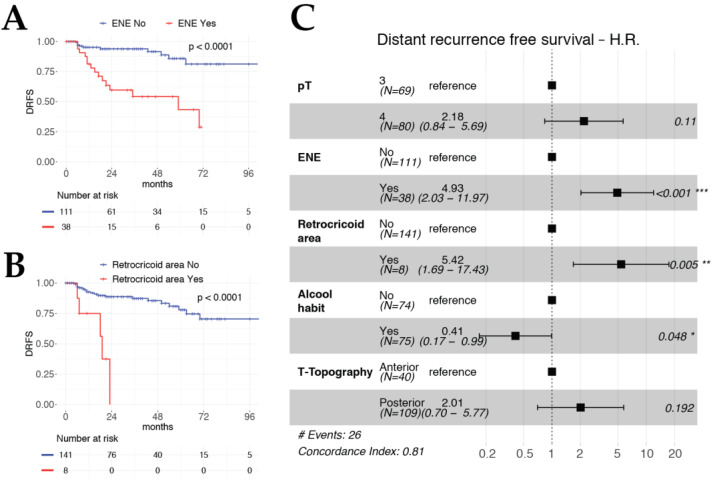
Kaplan–Meier curves for DRFS according to ENE status (**A**) and retro-cricoid area involvement (**B**). Forest plot of DRFS multivariable model (**C**).

Both multivariable OS (Figure 1) and DSS models (Figure 2) included pT and pN categories as significant and independent covariates, confirming their value as prognostic factors in the setting of TL for advanced LC. Interestingly, histopathologic evidence of ENE (HR 4.93, *p* < 0.001) and involvement of the retro-cricoid area (HR 5.42, *p* = 0.005) were strongly significant and independent worse prognostic factors for DRFS (Figure 3).

**Figure 4:**
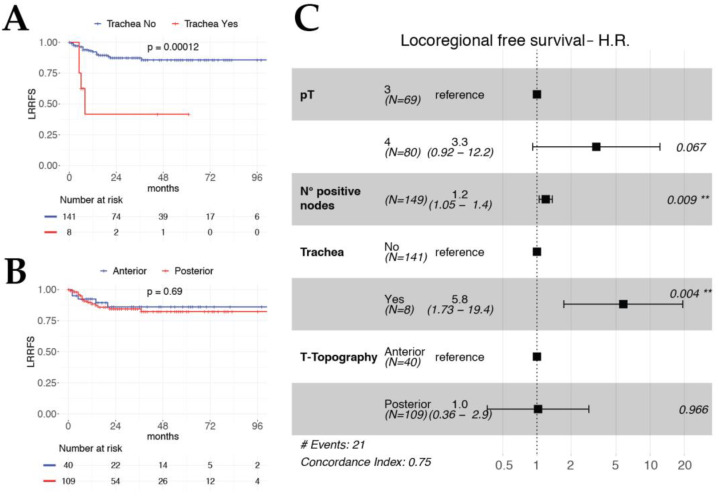
Kaplan–Meier curves for LRRFS according to the tracheal involvement (**A**) and T-topography (**B**). Forest plot of LRRFS multivariable model (**C**).

Considering the LRRFS multivariable model, the absolute number of positive lymph nodes, an emerging feature in the head and neck oncology field, was found to be an independent significant predictor (HR 1.2, *p* = 0.009). The same held true for tracheal rings involvement (HR 5.8, *p* = 0.004) (Figure 4). No significant interactions nor multicollinearity was observed in any model.

### 3.6. Search for the Best Cut-Off for Total Number of Positive Lymph Nodes for LRRFS Prognostic Factor

**Figure 5:**
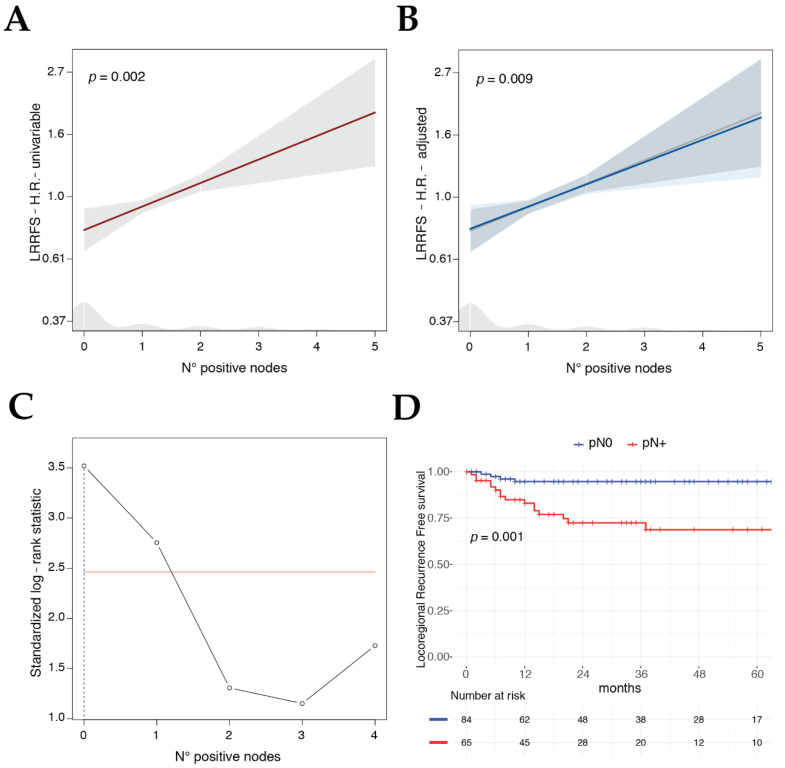
Plots showing the HR estimates and 95%CI (shadow area) of the number of histopathologic positive nodes derived from the univariable LRRFS Cox model (**A**) and from the multivariable one (**B**), adjusting the effect for pT category, tracheal involvement, and T-topography (gray line on the background refers to the univariable estimate). Plot of the standardized log-rank statistic, identifying 0 positive nodes as best cut-off for number of positive nodes for the prediction of LRRFS (**C**), red line indicating the adjusted significance level, vertical dotted line showing the best cut-off point. Kaplan–Meier curve of N status (N0 vs. N+) for LRRFS, p value adjusted for multiple tests showed (**D**).

The prognostic clinical relevance of the number of positive lymph nodes for LRRFS was consistent and stable considering it alone in a univariable model (Figure 5A), and after adjusting its effect (estimated by the HR) with the covariates included in the LRRFS multivariable model (Figure 5B). Identification of the total number of positive lymph nodes as one of the main prognostic features associated with loco-regional failure raised the question of if an optimal cut-off for this variable could be identified to better predict the ensuing outcomes in a dichotomic fashion. Applying the maximally selected log-rank statistics, a robust method that takes into account the multiplicity of tests, the best cut-off was zero positive nodes (*p* = 0.001, Figure 5C), splitting up pN0 vs. pN+ patients and confirming the relevance of this biologic and easy to measure feature (Figure 5D).

### 3.7. Clinical Significance of Histopathologic Tumor Thickness

**Figure 6:**
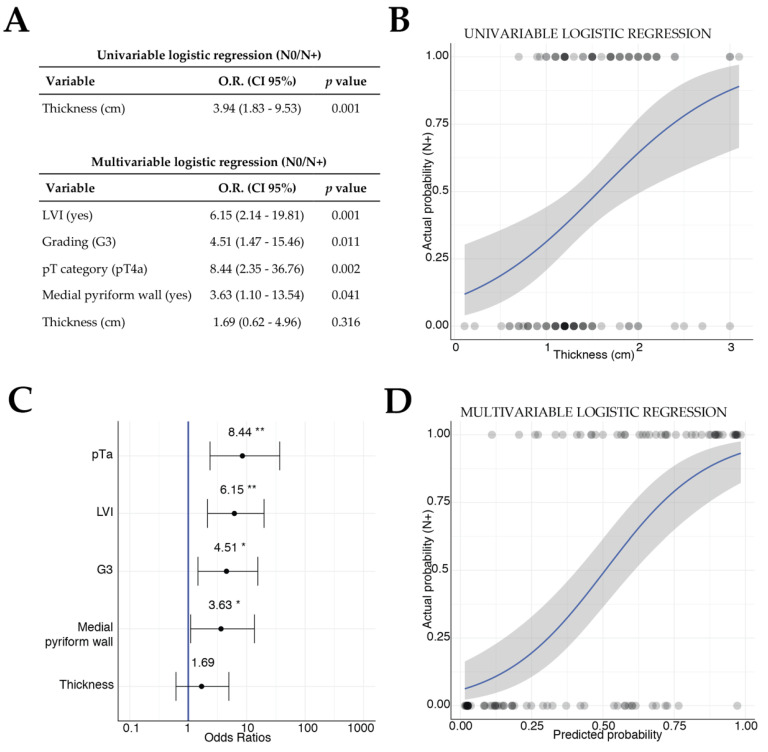
(**A**) Univariable logistic model including tumor thickness for the prediction of the N0 vs. N+ status and the multivariable one by stepwise variables selection. (**B**) Logistic function of the univariable logistic model including tumor thickness as covariate. (**C**) Forest plot of odds ratios and 95% CI of the multivariable logistic model for N status prediction. (**D**) Logistic function of the predicted probability in the multivariable logistic model against the actual probability of being N0 vs. N+. For logistic functions, 95% CI is showed with gray filling.

As the presence of metastatic lymph nodes was one of the main factors associated with loco-regional failure, histopathologic TT, available for 102 (68%) patients of the cohort, was investigated by logistic regression models for the prediction of the binary outcome pN0 vs. pN+. The univariable logistic model including just TT showed a significant association of this covariate with the presence of metastatic lymph nodes (OR 3.94, *p* = 0.001; Figure 6A,B). All available potential predictors, the covariates included in the survival analysis, were investigated in a multivariable logistic regression; the best model was built by a stepwise variable selection, keeping the TT inside the model as investigative variable (Figure 6A). Tumor category T4a (OR 8.44, *p* = 0.002), poor differentiation (OR 4.51, *p* = 0.011), presence of LVI (OR 6.15, *p* = 0.001), and involvement of the medial wall of the piriform sinus (OR 3.63, *p* = 0.041) were significantly and independently associated with the presence of lymph node metastases (Figure 6A,C,D), whereas TT in the multivariable model lost its association with the binary outcome N0 vs. N+ (*p* = 0.316) (Figure 6A,C).

## 4. Discussion

The potential for long-term survival in patients with advanced LC is nowadays significant and, consequently, the choice of the most adequate treatment option is of paramount value for optimizing cancer control, functional outcomes, and residual quality of life. Contemporary advances in endoscopic and radiologic diagnostic tools have allowed the scientific community to clearly understand that laryngeal CRT protocols should be reserved to highly selected patients with low-volume cancers, preserved airway patency and proper swallowing function, without the need for pre-treatment tracheostomy or feeding tube, limited cartilage destruction, and who can tolerate the toxicity of CHT associated with RT [4]. Following these recommendations, the population that ideally should undergo CRT protocols would result in a small fraction of T3-T4 LC patients. On the other hand, even OPHLs should be preferably reserved for patients <70 years of age who are neurologically intact and able to complete successful postoperative swallowing rehabilitation (a forecast so far left to the good, but largely fallible, clinical judgement of physicians), with good cardio-pulmonary function, and tumors not extending too far posteriorly and/or massively involving and ankylosing one crico-arytenoid unit [18]. Considering all these caveats and selection criteria, it is clear that TL still maintains a fundamental role in management of advanced LC. In this respect, the updated guidelines of ASCO rightly recommended TL for patients with large-volume T4 and/or poor pre-treatment laryngeal functions, since such a mutilating surgical procedure has nonetheless shown to be associated with better survival and, surprisingly, even superior quality of life compared to CRT or RT alone [4]. Facing this highly heterogeneous population of advanced LCs, and the even more differentiated gamma of therapeutic options available, each with its pros and cons, the relative paucity of information about which subgroup of patients might preferably benefit from primary OP protocols vs. upfront TL is definitively worrisome [19].

As a contribution to this topic, two recent studies have been published claiming that T-topography (distinguished in anterior vs. posterior involvement of the PGS) could represent a significant prognosticator in patients to be treated by OPHLs for T3–T4 LCs [11,12]. By contrast, in the present retrospective study focused on patients treated by upfront TL, we did not find a similar significant difference in survival outcomes comparing tumors with anterior vs. posterior laryngeal compartmentalization. The 5-year OS and DSS for anterior vs. posterior tumors, in fact, did not significantly differ at either univariable (*p* = 0.252 and *p* = 0.571, respectively) or multivariable analysis (*p* = 0.228 and *p* = 0.438, respectively). This should not represent a major source of disappointment if one considers that TL is able to radically remove the entirety of either an anterior or posterior advanced LC, possibly flattening the discrepancies in biological behavior and pathways of spread observed for more conservative surgical options such as TLM and OPHLs. Therefore, the concept of anterior vs. posterior laryngeal compartmentalization, while useful in deciding which lesions can be successfully managed by OP approaches, loses its appeal when considering survival after TL. On the other hand, pT category as currently defined by the 8th Edition of the TNM staging system maintains its role as a significant prognosticator for both 5-year OS and DSS estimates, as confirmed at multivariable analysis (*p* = 0.004 and *p* = 0.033, respectively), with values of 83% and 88% for pT3, and 47% and 65% for pT4a, respectively (Table S1).

Apart from the T issue, it is well established that, in advanced LCs, even N category plays a crucial role in treatment selection. In line with this, our data clearly show the survival impact of different nodal diseases (at OS and DSS univariable analysis, HR ranged respectively from 1.64 and 2.90 for pN1 to 4.72 and 6.14 for pN3 compared to the pN0 category). Moreover, as confirmation of the recent emphasis given to the ENE by the 8th Edition of the TNM staging system, its role as a negative prognosticator was confirmed at OS and DSS univariable analysis, with an HR of 3.23 and 3.57 compared to lymph nodes without ENE. Therefore, considering the last edition of the TNM staging system, it seems to efficiently stratify survival according to neck status, as also confirmed by a large study from the MD Anderson Cancer Center where node positive disease at presentation was associated with increased overall mortality (*p* < 0.0001) [20]. It is clear that patients with higher nodal category perform poorly regardless of treatment modality, and this poses a challenging problem particularly for advanced LC. In this scenario, while CRT is associated with worse OS compared to TL in some patients with T4 disease, no difference is seen among patients with T3 LC with minimal cartilage erosion, regardless of N status [21]. Dissecting these specific patients accurately, Choi at al. underlined that the survival improvement offered by primary surgery in T4 disease was significant only in N0-N1 patients, and not in those with higher neck categories [22]. In this cohort of patients, in fact, the survival benefit of TL was lost, whereas the performance of CRT remained stable between the T4N0-1 and T4N2-3 scenarios. This topic was further addressed by Patel et al. in a recent national database analysis, underling that there are no differences in survival between surgical (TL and OPHL) and non-surgical approaches (CRT) for non-T4 lesions with low nodal burden, while non-T4 tumors with high nodal burden benefit more from CRT. Nevertheless, it was pointed out that TL remains advantageous in patients with T4 LC [19]. According to these findings, adequate local control is more critical in patients with limited nodal involvement, as they have a relatively lower risk of distant metastasis, while patients with higher N categories are more exposed to distant failure. In this setting, patients with advanced nodal involvement might benefit more, starting at the beginning, from systemic therapeutic regimens that aim to control distant metastases [22].

In this regard, our investigation introduces another valuable piece of information: analyzing LRRFS, in fact, the impact of the number of tumor deposits in neck nodes was a detrimental prognostic factor at univariable analysis with an HR of 1.2 (*p* = 0.002), meaning an increase in risk of 20% for each positive node detected. This result was also confirmed at multivariable analysis, as both the number of positive nodes (HR 1.2, *p* = 0.009) and tracheal involvement (HR 5.8, *p* = 0.004) were significant covariates associated with loco-regional failure, independently of the T category. In this respect, Choi et al. proposed a new classification system for laryngeal and hypopharyngeal tumors treated with surgery, including the number of positive nodes and showing better performance for OS and DSS prediction compared to both the 7th and 8th Editions of the TNM staging system [23]. Of note, the relationship between the number of positive nodes and LRRFS highlighted in our study, has not been previously reported. Nonetheless, these findings are in accordance with those reported in the literature for other head and neck tumors [24], even though this topic was mainly investigated in the context of oral cavity squamous cell carcinoma [25,26,27].

One of the novel findings of our study is the preliminary investigation of the effects of TT in LC. To the best of our knowledge, the literature has rarely addressed this aspect, and most of the attempts to date present several drawbacks. Hirano, almost 30 years ago, tried to investigate the depth of vocal muscle invasion to better understand the pathophysiological mechanism of vocal fold hypomobility, although he did not relate it with LC prognosis [28]. A recent study partly addressed this issue, demonstrating worse outcomes resulting from the histopathological finding of deep infiltration into vocal muscle in early LC, though not correlating the ensuing prognosis with a continuous linear measurement [29]. On the other hand, Yilmaz et al. analyzed the role of deep neoplastic invasion in 74 laryngeal specimens, finding a direct correlation between this parameter and survival: in their work, the authors evaluated the correlation between depth of invasion (DOI) and nodal disease, showing that the mean DOI for N0, N1, N2, and N3 categories were 6.33, 8.72, 9.54, and 5.53 mm, respectively [30]. In addition, Kiliç et al. analyzed 85 patients treated by partial laryngectomies, aiming to correlate the occurrence of nodal metastasis with DOI [31]. In their study it was reported the presence of positive nodes starting from a DOI of 4 mm and this correlation proved to be significant only from a DOI of 20 mm by applying the Chi-square test. Ye et al. retrospectively analyzed a cohort of 127 patients affected by hypopharyngeal (93 patients) and supraglottic LC (34 patients), finding that DOI correlated with the probability of nodal metastasis both at univariable and multivariable analysis and proposing 4.5 mm as cut-off value for elective neck dissection; anyway glottic LC was not addressed in their study [32].

When investigating DOI in LC, the anatomical complexity of the larynx, which has a peculiar microanatomical structure that varies consistently, should be considered [33]. Advanced tumors can easily subvert the already compacted microstructures inside the laryngeal box: these pathological changes together with the complex microanatomy of this organ may complicate the evaluation of DOI in LC, as no study so far reported a standardized method to measure laryngeal DOI. Our investigation consequently addressed the TT, easily available retrospectively from pathological reports, to study its correlation with the risk of nodal disease. Interestingly, even if univariate analysis showed an association between TT and the presence of nodal metastasis, the multivariable logistic model revealed that other pathologic features such as T category, grading, LVI, and involvement of the medial wall of piriform sinus were significant and independent predictors for the presence of nodal metastases, overcoming the relevance of TT (Figure 6). This result might not seem of great interest in primary T4a LC, since most patients receive simultaneous elective neck dissection anyway. However, if this topic would be further investigated taking into account a standardized DOI (as extensively demonstrated for oral cavity squamous cell carcinoma), the implications could be of paramount importance even for LC. In those patients where, according to the NCCN guidelines, indications for neck dissection are not clear or debatable [15], as in selected T3 glottic cancers treated by TLM, T1-small T2 supraglottic cancers treated by transoral approaches, and recurrent/persistent LC failed after CRT and previously staged as cN0 [34], this information might be of great help in choosing to electively treat in one-stage or not both the T and N sites. In fact, as previously demonstrated, very few T3 glottic cancers fail regionally [13,35], while the role of neck dissection after CRT failure in cN0 neck is still controversial, some authors supporting an aggressive policy [36] and others maintaining a more cautious attitude [37].

Lastly, we analyzed the impact of LC extension to different subsites and found that involvement of the retro-cricoid area is a strong predictor for distant failure, especially at multivariable analysis (HR 5.42, *p* = 0.005). The best way to look at this finding is probably to compare it with what has been described for primary retro-cricoid hypopharyngeal carcinoma, frequently presenting with positive nodal disease and usually reported to have a poor prognosis with a 5-year OS ranging from 20% to 52% [38,39]. This type of localization, together with the piriform sinus, as pointed out in a large retrospective series [40], is prone to distant metastasis with a 17.2% rate. LC involving the post-cricoid area probably acquire a behavior similar to that of hypopharyngeal cancers, characterized by richer lymphatic drainage and a higher rate of nodal metastasis. As demonstrated by the authors, the incidence of distant disease is also directly related to positive lymph nodes, increasing the risk 3-fold [40]. Finally, at multivariable analysis, LC tracheal extension was shown to heavily affect LRRFS (HR 5.8, *p* = 0.004). In fact, achieving negative margins at this level is more challenging if the surgeon wants to preserve enough tracheal rings to tailor a good stoma and place the tracheo-esophageal speech prosthesis without risking locating it into the thoracic esophagus. Moreover, lymphatic drainage of the subglottis and trachea is prominent and may explain the rapid tumor spread observed when these locations are involved [41].

The retrospective nature of this study represents its main limit. More specifically, the addition of PORT and chemotherapy was discussed for every specific case at the MDT and cannot be fully standardized as it would be in a prospective randomized study. However, in pN1 LSCC, based on our results and some emerging evidence, even if it is not strictly indicated by the NCCN Guideline, the use of PORT might be considered. To the best of our knowledge, despite no survival differences were seen in T3 N0 or T3 N+ comparing TL+ PORT and CRT, the role of PORT is controversial in the subgroup of surgically treated T3N1, even retrieving the data from the largest and most comprehensive population-based studies [21,42]. We hope that further study will clarify if this very specific subset of patients might benefit or not of adjuvant treatment, or even curative RT/CRT. In such setting, more interesting pathological factors could be studied as the lymph-node size, the micro vs. macro ENE and the laryngeal DOI, to advocate the need for adjuvant treatment.

## 5. Conclusions

The data presented in this retrospective analysis support the hypothesis that laryngeal compartmentalization has no impact on survival in patients treated by upfront TL, but, most likely, it is a useful tool to identify ideal and unfavorable candidates for OP strategies. On the other hand, our findings demonstrate that the 8th Edition of the AJCC UICC TNM staging system is a robust prognosticator for advanced LC in terms of both T and N categories. Combining this information with previous reports, we might be able to better refine the decision-making process between CRT and upfront TL for advanced LC.

The retrospective nature of our study has inherent limitations that should be ideally overcome by a large scale, prospective study of surgically treated LC, in order to thoroughly investigate the value of TT and DOI for all disease categories, trying to find a correlation of histopathologic data with those obtainable in the pre-treatment setting by radiologic imaging. Lastly, a valuable topic to be investigated further is the impact of T-topography and laryngeal compartmentalization in patients to be managed by CRT as an alternative to OP surgery and TL.

## Figures and Tables

**Table 1:** Clinical and pathologic characteristics of the cohort.

Variables	Category	No.	%
Gender	Female	23	15.4
Smoking habit	Yes	142	95.3
Alcohol habit	Yes	75	50.3
Arytenoid fixation	Yes	90	60.4
Medial piriform sinus wall involvement	Yes	40	26.8
Radiologic posterior PGS involvement	Yes	70	47
T-topography	Posterior	109	73.2
Supraglottic involvement	Yes	122	81.9
Subglottic involvement	Yes	84	56.4
Base of tongue involvement	Yes	11	7.4
Retro-cricoid area involvement	Yes	8	5.4
Thyroid cartilage involvement	Inner cortex	30	20.1
	Full thickness	35	23.5
Cricoid cartilage involvement	Yes	36	24.2
Tracheal involvement	Yes	8	5.4
Cervical soft tissues involvement	Yes	50	33.6
Thyroid gland involvement	Yes	8	5.4
pT category	pT3	69	46.3
	Anterior pT3	19	12.7
	Posterior pT3	50	33.6
	pT4a	80	53.7
	Anterior pT4a	21	14.1
	Posterior pT4a	59	39.6
pN category	pN1	11	7.4
	pN2a	10	6.7
	pN2b	14	9.4
	pN2c	3	2
	pN3b	28	18.8
ENE	Yes	38	25.5
Surgical margins	Close	19	12.8
	Positive	11	7.4
Grading	G1	8	5.4
	G3	52	34.9
PNI	Yes	80	53.7
LVI	Yes	74	49.7
Treatments	S+PORT	72	48.3
	S+POCRT	17	11.4

Legend: ENE, extranodal extension; PNI, perineural invasion; LVI, lympho-vascular invasion; S, surgery; PORT, postoperative radiotherapy; POCRT, postoperative chemoradiotherapy.

**Table 2:** Univariable overall (OS) and disease specific survival (DSS) analysis (significant variables for at least one outcome are reported, while additional variables analyzed are reported in Table S2).

Variables	Category	OS			DSS		
		HR	CI95	*p*	HR	95% CI	*p*
T-topography	Posterior	1.51	(0.75–3.06)	0.252	1.28	(0.54–3.04)	0.571
pT category	pT4a	4.26	(2.03–8.96)	<0.001 *	5.98	(2.05–17.43)	0.001 *
pN category	pN1	1.64	(0.47–5.77)	0.440	2.90	(0.56–14.95)	0.204
	pN2a	3.71	(1.40–9.79)	0.008 *	8.88	(2.70–29.15)	<0.001 *
	pN2b	3.45	(1.30–9.14)	0.013 *	7.36	(2.12–25.54)	0.002 *
	pN2c	2.30	(0.30–17.66)	0.424	5.68	(0.66–48.79)	0.113
	pN3b	4.72	(2.22–10.03)	<0.001 *	6.14	(1.99–18.93)	0.002 *
ENE	Yes	3.23	(1.77–5.90)	<0.001 *	3.57	(1.67–7.63)	0.001 *
No. of positive nodes		1.16	(1.07–1.27)	0.001 *	1.17	(1.05–1.30)	0.004 *
Thyroid cartilage	Inner Cortex	1.09	(0.48–2.48)	0.845	1.27	(0.43–3.72)	0.661
	Full thickness	2.44	(1.27–4.71)	0.008 *	3.26	(1.41–7.57)	0.006 *
Soft tissues involvement	Yes	2.05	(1.13–3.73)	0.018 *	2.56	(1.19–5.50)	0.016 *
Retro-cricoid areainvolvement	Yes	2.88	(1.01–8.17)	0.047 *	4.80	(1.62–14.17)	0.005 *
Treatment	S+PORT	2.06	(1.01–4.19)	0.047 *	2.82	(1.03–7.67)	0.043 *
	S+POCRT	3.26	(1.25–8.56)	0.016 *	4.74	(1.35–16.60)	0.015 *

**Legend:** OS, overall survival; DSS, disease-specific survival; HR, hazard ratio; CI95, 95% confidence interval; ENE, extranodal extension; S, surgery; PORT, postoperative radiotherapy; POCRT, postoperative chemoradiotherapy; *Bold-italic, statistically significant results.

**Table 3:** Univariable loco-regional recurrence-free survival (LRRFS) and distant recurrence-free survival (DRFS) analysis (significant variables for at least one outcome are reported, while additional variables analyzed are reported in Table S3).

Variables	Category	LRRFS			DRFS		
		HR	95% CI	*p*	HR	95% CI	*p*
T-topography	Posterior	1.23	(0.45–3.39)	0.689	1.83	(0.68–4.88)	0.230
pT category	pT4a	5.47	(1.60–18.69)	0.007 *	3.49	(1.39–8.76)	0.008 *
pN category	pN1	3.99	(0.73–21.79)	0.110	1.07	(0.13–8.71)	0.949
	pN2a	6.47	(1.45–28.93)	0.015 *	6.64	(2.10–20.99)	0.001 *
	pN2b	5.03	(1.12–22.52)	0.035 *	2.30	(0.47–11.13)	0.301
	pN2c	7.10	(0.79–63.67)	0.080	-	-	-
	pN3b	6.13	(1.79–20.97)	0.004 *	5.99	(2.26–15.88)	<0.001 *
ENE	Yes	3.26	(1.36–7.85)	0.008 *	5.61	(2.51–12.52)	<0.001 *
No. of positive nodes		1.20	(1.07–1.36)	0.002 *	1.26	(1.13–1.41)	<0.001 *
Surgical margins	Close	1.77	(0.58–5.38)	0.314	3.58	(1.44–8.91)	0.006 *
	Positive	1.76	(0.40–7.75)	0.457	3.05	(0.88–10.65)	0.080
LVI	Yes	1.55	(0.63–3.79)	0.340	2.52	(1.09–5.86)	0.032 *
Treatment	S+PORT	2.13	(0.76–5.91)	0.148	2.63	(0.95–7.28)	0.063
	S+POCRT	0.75	(0.09–6.42)	0.792	5.04	(1.45–17.55)	0.011 *
Tracheal involvement	Yes	6.65	(2.18–20.27)	0.001 *	3.70	(1.09–12.51)	0.036 *
Retro-cricoid area involvement	Yes	3.64	(1.06–12.47)	0.040 *	8.39	(2.96–23.81)	<0.001 *

**Legend:** LRRFS, loco-regional recurrence-free survival; DRFS, distant recurrence-free survival; HR, hazard ratio; 95% CI, 95% confidence interval; ENE, extranodal extension; LVI, lympho-vascular invasion; S, surgery; PORT, postoperative radiotherapy; POCRT, postoperative chemoradiotherapy; *Bold-italic, statistically significant results.

## Supplementary Materials

The following are available online at https://www.mdpi.com/2072-6694/12/8/2241/s1. Table S1: Survival estimates at 2 years (2-y) and 5 years (5-y) for main clinical interest covariates. Table S2: Univariable OS and DSS analysis, additional variables. Table S3: Univariable LRRFS and DRFS analysis, additional variables. Figure S1: Kaplan–Meier curves of univariable overall survival (OS) and disease-specific survival (DSS) considering radiologic posterior PGS involvement and arytenoid motility.
