# Supplementary material for: Laryngeal Compartmentalization Does Not Affect the Prognosis of T3-T4 Laryngeal Cancer Treated by Upfront Total Laryngectomy
Source: Cancers (Basel). 2020 Aug 11;12(8):2241. doi: 10.3390/cancers12082241 (PMC7463701; doi:10.3390/cancers12082241)
Supplement: Supplementary file 1 [file cancers-12-02241-s001.pdf]

cancers

MDPI

Article

Laryngeal Compartmentalization Does Not Affect the Prognosis of T3-T4 Laryngeal Cancer Treated by Upfront Total Laryngectomy

FilippoMarchi,FrancescoMissale,ClaudioSampieri,MartaFilauro,AndreaIandelli,GiampieroParrinello,FabiolaIncandela,LudwigE.Smeele,MichielW.M.vandenBrekel,FrancescaDelBon,PieroNicolai,CesarePiazzaandGiorgioPeretti

Supplementary materials:

TableS1. Survival estimates at 2 (2-y) and 5years (5-y) for the main covariates of clinical inter**est.**

			OS	DSS	DRFS	LRRFS
		Years	Estimate(CI95)	Estimate(CI95)	Estimate(CI95)	Estimate(CI95)
	All					
		2-y	0.77(0.69–0.83)	0.84(0.76–0.89)	0.85(0.77–0.9)	0.85(0.77–0.9)
		5-y	0.63(0.51–0.72)	0.75(0.64–0.83)	0.75(0.62–0.83)	0.83(0.75–0.89)
	Tcategory					
pT3		2-y	0.91(0.8–0.96)	0.96(0.86–0.99)	0.94(0.84–0.98)	0.95(0.84–0.98)
		5-y	0.83(0.65–0.92)	0.88(0.68–0.96)	0.86(0.68–0.95)	0.95(0.84–0.98)
pT4a		2-y	0.66(0.54–0.76)	0.74(0.62–0.83)	0.76(0.64–0.85)	0.76(0.64–0.85)
		5-y	0.47(0.32–0.6)	0.65(0.5–0.77)	0.64(0.45–0.78)	0.74(0.6–0.83)
	Nstatus					
N0		2-y	0.86(0.76–0.92)	0.93(0.84–0.97)	0.94(0.86–0.98)	0.95(0.87–0.98)
		5-y	0.77(0.63–0.86)	0.9(0.78–0.96)	0.88(0.74–0.95)	0.95(0.87–0.98)
N+		2-y	0.66(0.52–0.77)	0.73(0.59–0.83)	0.72(0.58–0.83)	0.72(0.58–0.83)
		5-y	0.46(0.29–0.61)	0.57(0.38–0.72)	0.57(0.36–0.73)	0.69(0.53–0.8)
	Arytenoidfixation					
No		2-y	0.81(0.66–0.9)	0.87(0.73–0.94)	0.92(0.79–0.97)	0.88(0.74–0.94)
		5-y	0.73(0.55–0.85)	0.79(0.6–0.89)	0.81(0.59–0.92)	0.88(0.74–0.94)
Yes		2-y	0.76(0.65–0.84)	0.82(0.71–0.89)	0.81(0.69–0.89)	0.84(0.73–0.91)
		5-y	0.56(0.4–0.69)	0.74(0.59–0.84)	0.71(0.54–0.82)	0.81(0.69–0.89)
	PosteriorPGS					
No		2-y	0.77(0.65–0.85)	0.85(0.74–0.92)	0.89(0.79–0.95)	0.83(0.72–0.9)
		5-y	0.66(0.51–0.77)	0.8(0.67–0.89)	0.82(0.65–0.91)	0.83(0.72–0.9)
Yes		2-y	0.77(0.64–0.86)	0.82(0.69–0.9)	0.79(0.66–0.88)	0.88(0.76–0.94)
		5-y	0.58(0.38–0.73)	0.66(0.44–0.81)	0.66(0.46–0.8)	0.84(0.7–0.92)
	Tcategory					
pT3ant		2-y	0.89(0.62–0.97)	1	1	0.87(0.58–0.97)
		5-y	0.81(0.51–0.94)	0.91(0.51–0.99)	1	0.87(0.58–0.97)
pT3post		2-y	0.92(0.78–0.97)	0.95(0.8–0.99)	0.92(0.77–0.97)	0.98(0.85–1)

	5-y	0.85(0.59–0.95)	0.87(0.59–0.96)	0.8(0.56–0.92)	0.98(0.85–1)
pT4aant	2-y	0.72(0.44–0.87)	0.77(0.5–0.91)	0.87(0.58–0.97)	0.84(0.58–0.95)
	5-y	0.72(0.44–0.87)	0.77(0.5–0.91)	0.73(0.32–0.92)	0.84(0.58–0.95)
pT4apost	2-y	0.64(0.49–0.75)	0.72(0.58–0.83)	0.72(0.57–0.83)	0.73(0.58–0.84)
	5-y	0.38(0.22–0.55)	0.61(0.43–0.75)	0.63(0.44–0.77)	0.7(0.53–0.81)
T-topography					
Anterior	2-y	0.8(0.63–0.9)	0.89(0.72–0.96)	0.94(0.78–0.98)	0.86(0.69–0.94)
	5-y	0.76(0.56–0.87)	0.83(0.63–0.93)	0.85(0.56–0.96)	0.86(0.69–0.94)
Posterior	2-y	0.76(0.66–0.83)	0.82(0.72–0.89)	0.81(0.71–0.88)	0.84(0.75–0.91)
	5-y	0.57(0.43–0.69)	0.71(0.56–0.82)	0.7(0.56–0.81)	0.82(0.72–0.89)

Table S2. Univariable OS and DSS analysis, additional variables.

Variables	Category	OS			DSS		
		HR	CI95	p	HR	CI95	*p*
**Age**		1.03	(0.99–1.06)	0.112	1.03	(0.99–1.07)	0.176
Gender	F	0.67	(0.27–1.72)	0.408	0.66	(0.20–2.18)	0.492
Smoke	Yes	0.60	(0.18–1.95)	0.395	1.19	(0.16–8.82)	0.862
Alcohol	Yes	0.91	(0.50–1.65)	0.764	0.90	(0.42–1.92)	0.788
Arytenoidfixation	Yes	1.37	(0.73–2.59)	0.328	1.20	(0.54–2.66)	0.657
PosteriorPGS	Yes	1.08	(0.59–1.98)	0.791	1.30	(0.61–2.80)	0.497
Supraglottis	Yes	1.56	(0.66–3.71)	0.310	1.08	(0.41–2.85)	0.879
Subglottis	Yes	0.76	(0.42–1.38)	0.365	0.94	(0.44–2.02)	0.879
Medialpiriformsinus	Yes	1.50	(0.81–2.81)	0.201	2.02	(0.93–4.35)	0.074
Baseoftongue	Yes	1.52	(0.59–3.92)	0.391	1.00	(0.23–4.26)	0.995
Trachea	Yes	2.33	(0.83–6.54)	0.110	2.90	(0.87–9.69)	0.084
Grading	G1	0.63	(0.09–4.70)	0.655	1.03	(0.14–7.88)	0.974
	G3	1.42	(0.78–2.58)	0.257	1.29	(0.59–2.80)	0.522
Margins	Close	1.68	(0.77–3.66)	0.195	2.12	(0.84–5.37)	0.112
	Positive	1.53	(0.46–5.04)	0.484	1.67	(0.39–7.23)	0.494
PNI	Yes	0.82	(0.46–1.49)	0.524	1.19	(0.55–2.57)	0.652
LVI	Yes	1.41	(0.77–2.59)	0.260	2.16	(0.96–4.83)	0.061
Cricoidcartilage	Yes	1.25	(0.65–2.40)	0.495	1.76	(0.80–3.85)	0.159
Thyroidgland	Yes	0.71	(0.17–2.94)	0.635	0.57	(0.08–4.21)	0.580
Tcategory	pT3posterior	1.00	(0.25–3.99)	0.997	1.50	(0.16–14.39)	0.727
	pT4aanterior	2.99	(0.77–11.66)	0.115	7.12	(0.85–59.29)	0.070
	pT4aposterior	4.77	(1.44–15.83)	0.011	8.34	(1.10–63.10)	0.040

Table S3. LRRFS and DRFS analysis, additional variables.

Variables	Category	LRRFS			DRFS		
		HR	CI95	p	HR	CI95	p
Age		1.00	(0.95–1.04)	0.866	1.01	(0.97–1.05)	0.640
Gender	F	1.36	(0.45–4.06)	0.586	0.99	(0.34–2.90)	0.990
Smoke	Yes	-	-	-	1.00	(0.13–7.40)	0.998
Alcohol	Yes	1.04	(0.43–2.51)	0.930	0.73	(0.33–1.61)	0.434
Arytenoidfixation	Yes	1.47	(0.56–3.87)	0.435	1.61	(0.68–3.79)	0.276
PosteriorPGS	Yes	0.80	(0.33–1.96)	0.630	1.82	(0.82–4.03)	0.141
Supraglottis	Yes	0.68	(0.25–1.86)	0.448	0.98	(0.37–2.60)	0.961
Subglottis	Yes	1.36	(0.54–3.41)	0.512	0.83	(0.38–1.83)	0.650
Medialpiriformsinus	Yes	1.43	(0.57–3.57)	0.450	1.69	(0.74–3.83)	0.211
Baseoftongue	Yes	1.81	(0.42–7.83)	0.425	0.53	(0.07–3.93)	0.534
Grading	G1	1.13	(0.15–8.77)	0.906	1.09	(0.14–8.35)	0.938
	G3	1.19	(0.48–2.95)	0.712	1.52	(0.68–3.40)	0.302
PNI	Yes	0.86	(0.36–2.06)	0.729	1.07	(0.48–2.36)	0.871
Thyroidcartilage	InnerCortex	1.45	(0.48–4.32)	0.508	1.00	(0.36–2.82)	0.995
	Fullthickness	1.74	(0.62–4.88)	0.295	1.62	(0.64–4.07)	0.308
Softtissuesinvolvement	Yes	2.12	(0.88–5.08)	0.094	1.95	(0.89–4.27)	0.097
Thyroidgland	Yes	0.93	(0.12–6.98)	0.947	2.10	(0.62–7.06)	0.230
Tcategory	pT3posterior	0.21	(0.02–2.36)	0.208	2.32	(0.27–19.87)	0.443
	pT4aanterior	1.53	(0.26–9.19)	0.639	4.47	(0.50–40.09)	0.181
	pT4aposterior	2.82	(0.64–12.44)	0.170	7.64	(1.01–58.10)	0.049

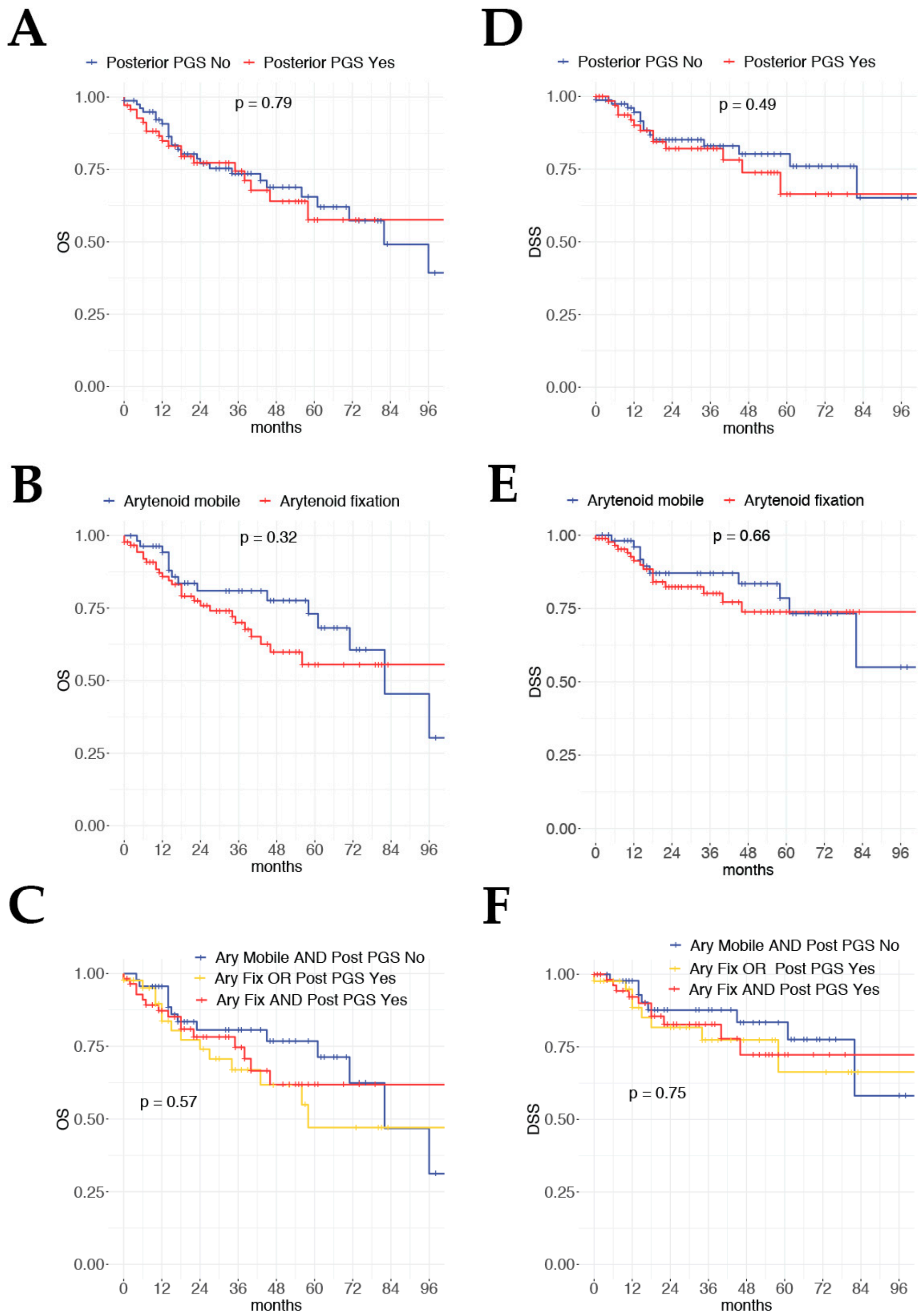

Figure S1. Kaplan-Meier curves of univariable OS (**A, B, C**) and DSS (**D, E, F**) considerin gradiologic posterior PGS involvement and arytenoid motility or a combination of them.

©2020bytheauthors.Submittedforpossibleopenaccesspublicationunderthetermsandconditionsoft eCreativeCommonsAttribution(CCBY)license(http://creativecommons.org/licenses/by/4.0/).
